# Phylogenetic Investigation of Peptide Hormone and Growth Factor Receptors in Five Dipteran Genomes

**DOI:** 10.3389/fendo.2013.00193

**Published:** 2013-12-16

**Authors:** Kevin J. Vogel, Mark R. Brown, Michael R. Strand

**Affiliations:** ^1^Department of Entomology, The University of Georgia, Athens, GA, USA

**Keywords:** GPCR, receptor tyrosine kinase, guanylyl cyclase, serine/threonine kinase, neuropeptide, protein hormone

## Abstract

Peptide hormones and growth factors bind to membrane receptors and regulate a myriad of processes in insects and other metazoans. The evolutionary relationships among characterized and uncharacterized (“orphan”) receptors can provide insights into receptor-ligand biology and narrow target choices in deorphanization studies. However, the large number and low sequence conservation of these receptors make evolutionary analysis difficult. Here, we characterized the G-protein-coupled receptors (GPCRs), receptor guanylyl cyclases (RGCs), and protein kinase receptors (PKRs) of mosquitoes and select other flies by interrogating the genomes of *Aedes aegypti, Anopheles gambiae, Culex quinquefasciatus, Drosophila melanogaster*, and *D. mojavensis*. Sequences were grouped by receptor type, clustered using the program CLANS, aligned using HMMR, and phylogenetic trees built using PhyML. Our results indicated that PKRs had relatively few orphan clades whereas GPCRs and RGCs had several. In addition, more than half of the Class B secretin-like GPCRs and RGCs remained uncharacterized. Additional studies revealed that Class B GPCRs exhibited more gain and loss events than other receptor types. Finally, using the neuropeptide F family of insect receptors and the neuropeptide Y family of vertebrate receptors, we also show that functional sites considered critical for ligand binding are conserved among distinct family members and between distantly related taxa. Overall, our results provide the first comprehensive analysis of peptide hormone and growth factor receptors for a major insect group.

## Introduction

Neuropeptides, protein hormones, and growth factors regulate many processes by binding to three types of membrane receptors: G-protein-coupled receptors (GPCRs), receptor guanylyl cyclases (RGCs), and protein kinase receptors (PKRs). We use the term peptide hormones to encompass both larger protein hormones released into the hemolymph by glands, neuroendocrine cells, and midgut endocrine cells, and neuropeptides that function as neurotransmitters and modulators within the nervous system. The greatest number and diversity of peptide hormones are bound by GPCRs, whereas known growth factors interact almost exclusively with PKRs. All of these receptor proteins are post-translationally modified and transported to the cell membrane. They encode an extracellular amino (N)-terminal region, one or more transmembrane spans, and an intracellular carboxyl (C)-terminal region that transduces ligand binding through specific signaling and amplification pathways. However, most GPCRs that bind peptide hormones reside as monomers in the cell membrane, whereas most RGCs and PKRs form dimers prior to or during ligand binding (1–3).

The genomes of several insects and related arthropods (*Aedes aegypti, Anopheles gambiae, Apis mellifera, Bombyx mori, Drosophila melanogaster, Nasonia vitripennis, Tribolium castaneum, Tetranychus urticae*, and *Daphnia pulex*) have been sequenced and their peptide hormone genes analyzed (4–15). GPCRs known or predicted to bind specific peptide hormones have also been annotated for *D. melanogaster* (7, 16), *Ap. mellifera* (17), *T. castaneum* (18), *B. mori* (19, 20), and *T. urticae* (13). These studies provide important insights about peptide hormone and GPCR diversity among arthropods while also shedding light on the evolutionary history and function of certain genes. These analyses also indicate that several peptide hormones and GPCRs remain “orphans” (6, 9, 16, 18, 21, 22). In some cases, one-to-one orthologs of known ligand-receptor pairs in the genomes of related species have led to predicted pairing to an uncharacterized receptor (23). For others though, ligand-receptor relationships remain unclear because gene duplication or loss events either create uncertainties about the functional homology of in- and out-paralogs, or have resulted in clades that contain no characterized orthologs (7, 21, 24, 25). We also note that no lists have been published for RGCs or PKRs and their associated ligands in any insect.

Our interests primarily focus on mosquitoes (Diptera: Culicidae), which are critically important insects because of their ability to vector several disease-causing pathogens to humans and other mammals. Peptide hormones and growth factors are key regulators of many physiological processes in mosquitoes that affect disease transmission. Three vector species of mosquitoes have been sequenced: *An. gambiae* (26), *Ae. aegypti* (27), and *Culex quinquefasciatus* (28). Annotation of the *An. gambiae* and *Ae. aegypti* genomes identify 35 and 43 peptide hormone genes respectively that are expressed in the nervous system, isolated glands, or midgut endocrine cells (9, 10). The function of several of these hormones is known (Table S1 in Supplementary Material). Homology-based searches were previously used to characterize the GPCR superfamily in *An. gambiae* (24) and analysis of the *Ae. aegypti* genome also identified some predicted peptide hormone GPCRs (27). No recent collation of GPCRs or other receptor types, however, is available.

Here, we analyzed the GPCRs, PKRs, and RGCs that bind peptide hormones and growth factors in mosquitoes and select other Diptera to discern phylogenetic patterns of receptor evolution. Our results provide a number of new insights including the identification of several orphan receptors. Ligand binding studies are the only means to deorphanize a given receptor definitively. However, our results could greatly assist deorphanization studies because they identify evolutionary relationships between receptors and thus narrow the spectrum of candidate ligands that a given orphan most likely binds (29, 30).

## Materials and Methods

### Identification of peptide hormone receptors

Peptide hormone receptors were identified from previously published surveys of *D. melanogaster* (7), *An. gambiae* (24), and *Ae. aegypti* (27). These sequences were downloaded from NCBI and used to plumb the genomes of *D. mojavensis* and *C. quinquefasciatus* using BLASTp. Each set of peptide hormone receptors from an organism was searched against the other genomes. The program HMMscan (31) was used to independently verify the completeness of our gene sets. Each genome was scanned using the Pfam models for rhodopsin-like GPCRs (“7tm_1,” PF00001), secretin-like GPCRs (“7tm_2,” PF00002), and protein kinases (“Pkinase,” PF00069) for RGCs and PKRs. These protein sequences were also used to search OrthoDB (32) for orthologs that may have been missed by our homology-based searches. The lists of receptors identified by Pfam searches were compared to the lists generated by BLAST searches. Genes were retained for further analysis if they were identified in Pfam searches or had a BLAST hit that had >50% amino acid identity with a known GPCR, RGC, or PKR in one of the examined genomes. In some instances, multiple annotated genes were found to encode parts of a single receptor. We used publicly available RNAseq data where available to join separate genes. In other cases, multiple predicted genes aligned to successive regions of orthologs in genomes with superior annotations (*D. melanogaster, An. gambiae*), and this was taken as evidence that the genes had been split improperly during gene prediction. The split genes were concatenated and used for downstream analysis. The complete list of receptors and accessions for each species used in our study is presented in Table S1 in Supplementary Material.

### Clustering of GPCR sequences

Attwood and Findlay (33) previously categorized GPCRs into six Classes (A–F) on the basis of shared sequence motifs and ligand binding affinities. Arthropod GPCRs reside in four of these classes: rhodopsin-like (Class A), secretin-like (Class B), metabotropic glutamate receptors (Class C), and frizzled/smoothened (Class F) (7, 13, 21, 34). Only Classes A and B contain receptors whose known or predicted ligands are peptide hormones. Our analysis likewise divided peptide hormone binding GPCRs into Class A rhodopsin-like GPCRs and Class B secretin-like GPCRs. We further analyzed the rhodopsin-like group using the CLANs program (35), which uses BLAST-search based similarity to create self-organizing maps (SOM). CLANs was run for 2000 rounds with an *e*-value cutoff of 10^−5^. Clusters were determined by bootstrap-based clustering with a minimum of 4 sequences per cluster and 500 rounds.

### Alignment and phylogenetic tree construction

For GPCRs, protein sequence alignments were initially attempted using MAFFT (36) and MUSCLE (37). Multiple parameters were tested but failed to produce acceptable alignments. Due to an improved ability to align divergent sequences with conserved domains, the program HMMalign (31) was used to align sequences using the Pfams mentioned previously. The highly variable N- and C-terminal regions of the proteins were removed by implementing the – “trim” option and poorly aligned regions were manually removed using Jalview (38). Alignments are included in the Files S1–S5 in Supplementary Material.

Phylogenetic trees were constructed from alignments using PhyML (39) on the LIRMM server. Parameters were as follows: substitution model was LG, proportion of invariable sites was 0.0, four substitution categories were used, initial trees were constructed using BIONJ, tree improvement was done through NNI, and trees were optimized for topology and branch length. Likelihood scores were computed using aBayes. Trees were visualized in FigTree v1.3.1. For the GPCR orphans, we arbitrarily numbered each clade of orphans and included the letter, A or B, to indicate which GPCR class they belonged to. RGC and PKR orphan clades are indicated as OR# and OGC#, respectively.

### Conservation of ligand binding domains

Deduction of receptor function from phylogenetic analysis is contingent upon conservation of the ligand binding region of the receptor and essential residues of the hormone. To examine whether functionally important regions of related receptors and ligands were conserved in divergent organisms, we examined neuropeptide F (NPF) in insects and its homologs in vertebrates, neuropeptide Y (NPY), pancreatic polypeptide (PP), and peptide YY (PYY). We aligned the NPF GPCRs from the five dipteran genomes with the NPY, PP, and PYY GPCRs from *Homo sapiens, Mus musculus*, and *Danio rerio* as described for the other GPCRs and visualized in Jalview (38). Previous targeted mutagenesis studies distinguished residues important for ligand binding (40–43) that were then identified and highlighted in the GPCR sequences.

## Results

### Database mining and phylogenetic analysis of dipteran receptors

Prior studies provide strong support for the monophyly of the Diptera while also showing that mosquitoes (Culicomorpha: Culicidae) are an early lineage that evolved ca. 225 Mya and drosophilids like *D. melanogaster* are a derived lineage (Ephydroidea: Drosophilidae) that emerged concurrently with other cyclorrhaphan flies 40–65 Mya (44). Prior studies also support the monophyly of the Culicidae, which consists of two subfamilies, the Anophelinae (as represented by *An. gambiae*) and the Culicinae (as represented by *Ae. aegypti* and *C. quinquefasciatus*) that diverged ca. 145–200 Mya (45, 46). We thus organized our study of peptide hormone and growth factor receptors to span both subfamilies of the Culicidae plus the phylogenetic breadth of the Diptera by including data from *D. melanogaster* and one other drosophilid (*D. mojavensis*). For each receptor group, we first summarize key features of our analysis.

We then address the receptors in specific clades and orphans of interest and the gene identification for the receptors in the five species. The known functions for peptide hormones in mosquitoes are summarized in Table S1 in Supplementary Material, along with the relevant references whereas the review by Nässel and Winther (58) provides an in depth summary of the function and signaling of peptide hormones in *D. melanogaster*. In addition, selected information and references for growth factors and their receptors and the RGCs characterized in *D. melanogaster* are provided in Table S1 in Supplementary Material. VectorBase or FlyBase accessions are also provided for all genes used in the study in Table S1 in Supplementary Material.

### Peptide hormone GPCRs

We focused on the GPCRs first because their interactions with peptide hormones have been more extensively examined in insects than those of RGCs and PKRs. GPCRs are 40–60 kDa proteins, which contain an extracellular N-terminal region, seven transmembrane α-helices that form a ligand binding pocket, and an intracellular C-terminal region that mediates signaling through interactions with G-proteins. G-proteins consist of different subunits that interact with other proteins to produce a variety of intracellular signaling molecules including cAMP, cGMP, and calcium.

**Figure 1:**
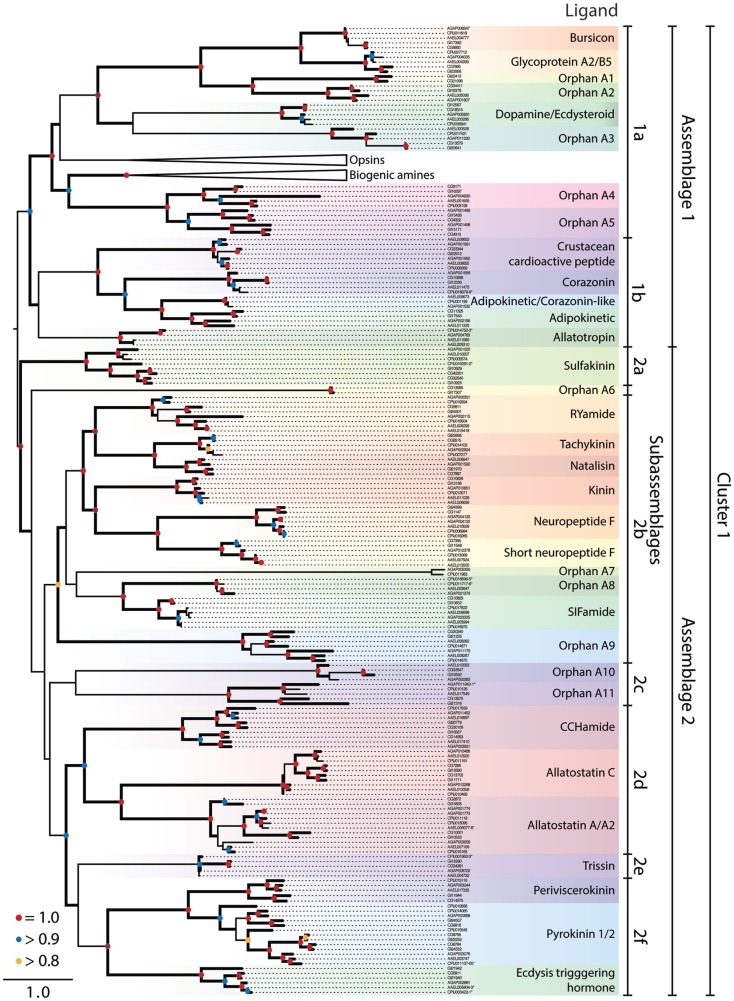
**Maximum-likelihood phylogeny of peptide hormone, Class A rhodopsin-like GPCRs in Cluster 1**. The phylogeny presented is based on the seven transmembrane repeat region of proteins from *Ae. aegypti, An. gambiae, C. quinquefasciatus, D. melanogaster*, and *D. mojavensis*. Entries are identified by their VectorBase or FlyBase ID and accession number prefixes that indicate species: CG#, *D. melanogaster*; GI#, *D. mojavensis*; AAEL#, *Ae. aegypti*; AGAP#, *An. gambiae*; CPIJ#, *C. quinquefasciatus*. Assemblages and sub-assemblages are indicated to the right of the phylogeny. Each monophyletic clade of characterized or orphan GPCRs is highlighted with a different color and named after characterized ligands, where known. Clades containing the opsin and biogenic amine GPCRs have been collapsed. Nodes with likelihood scores >0.8 are denoted by thickened lines. Dots at nodes indicate level of support: 0.8–0.9 (yellow), 0.9–0.99 (blue), 1 (red); instances of split gene annotations are denoted by the inclusive range of accession numbers and marked with an asterisk. Isoforms of proteins were initially included in the tree but were removed if the two forms had a branch length of 0, and the tree was midpoint rooted.

**Figure 2:**
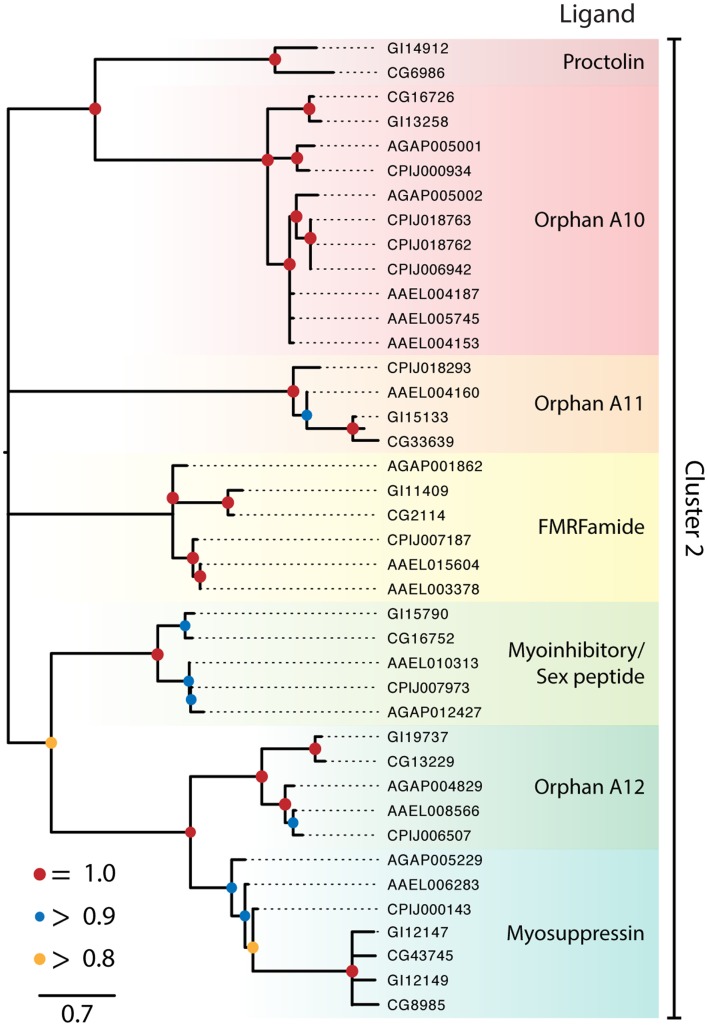
**Maximum-likelihood phylogeny of peptide hormone, Class A rhodopsin-like GPCRs in Cluster 2**. Phylogenies were generated and named as described in Figure 1, except that nodes with likelihood scores <0.8 have been collapsed to polytomies. Isoforms of proteins were initially included in the tree but were removed if the two forms had a branch length of 0 and the tree was midpoint rooted.

**Figure 3:**
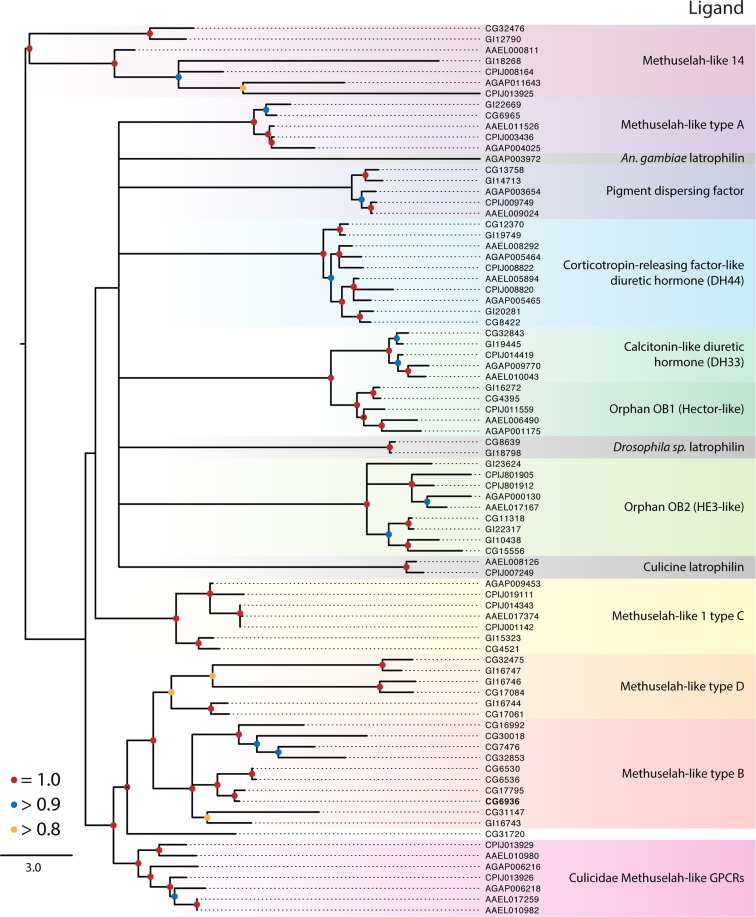
**Maximum-likelihood phylogeny of the Class B secretin GPCRs**. Phylogenies were generated and named as described in Figure 2. The latrophilin receptors (gray shading) contain only a single transmembrane repeat, resulting in a poor alignment and phylogenetic placement (see text). Using full-length latrophilin sequences resurrects the monophyly of this group of unusual GPCRs (data not shown). Methuselah clades were named according to Li et al. (126). The *D. melanogaster* Methuselah receptor that binds Stunted is highlighted in bold. The tree was rooted at the midpoint.

Most phylogenies generated previously for insect GPCRs utilized standard alignment algorithms (ClustalW or MegAlign) followed by neighbor-joining methods. Only rarely though have branch support values or other measures of robustness been reported (16). We initially used the full-length predicted amino acid sequence for each GPCR in our data set for tree building using the advanced alignment algorithm MAFFT. However, as found for vertebrates and other arthropods (17, 21, 30, 47), these approaches failed to produce suitable alignments due to extensive divergence in the N- and C-termini flanking the transmembrane domains of the predicted proteins (data not shown). We therefore used only the seven transmembrane α-helices for each GPCR followed by alignment using the domain-based algorithm HMMalign (31). This approach yielded suitable alignments for tree building. We sought to combine the previously divided GPCR subgroups as much as possible while retaining reasonable support at deep nodes within the trees. To achieve this, we used CLANS (35), which produced two distinct clusters (1 and 2) of Class A rhodopsin-like GPCRs with 352 and 41 proteins respectively (Table 1; Figures 1 and 2). Our analysis further indicated the Class B secretin-like GPCRs contained 82 proteins (Table 1; Figure 3).

**Table 1:** **Distribution of peptide hormone and growth factor receptors by type, species and characterization**.

	Class A GPCR Cluster 1	Class A GPCR Cluster 2	All Class A GPCR	Class B GPCR	PKR	RGC
Total genes	204	41	245	82	93	31
Clades	34	7	41	11	17	6
Characterized clades	23 (68%)	4 (57%)	27 (66%)	3 (27%)	15 (88%)	3 (50%)
Orphan clades	11 (32%)	3 (43%)	14 (34%)	8 (72%)	2 (12%)	3 (50%)
“In group” orphans	33 (13%)	1 (2.4%)	27 (11%)	0 (0%)	9 (10%)	0 (0%)
*Ae. aegypti* genes	39	9	48	13	22	7
*An. gambiae* genes	41	6	47	12	16	6
*C. quinquefasciatus* genes	40	9	49	16	18	6
*D. melanogaster* genes	37	8	45	24	16	7
*D. mojavensis* genes	38	8	46	17	17	5

### Class a rhodopsin-like GPCRs

Cluster 1 for the Class A rhodopsin-like GPCRs contained peptide hormone receptors, opsins, and biogenic amine GPCRs that our analysis divided into two monophyletic clades we term assemblages 1 and 2 (Figure 1). We further divided the GPCR assemblages into sub-assemblages of nested, well-supported monophyletic clades, denoted by a letter after the assemblage they belong to. Assemblage 1 consisted of two deeply divergent sub-assemblages, 1a and b (Figure 1). Sub-assemblage 1a contained the bursicon, glycoprotein A2/B5, and dopamine/ecdysteroid receptors, three interspersed orphan clades, OA13, as well as two deeply diverging orphan clades named OA4–5 (Figure 1). Sub-assemblage 1a also contained the opsins and biogenic amine GPCRs. Sub-assemblage 1b contained the receptors for crustacean cardioactive peptide (CCAP), corazonin, adipokinetic/corazonin-like peptide (ACP), adipokinetic hormone (AKH), and allatotropin. Assemblage 2 consisted of 25 peptide hormone receptors and 5 orphan clades, which formed the following monophyletic sub-assemblages: (2a) sulfakinin; (2b) RYamide, tachykinin, natalisin, kinin, NPF, and short NPF (sNPF), and SIFamide; (2c) orphan clades A10–11; (2d) CCHamide, allatostatin A and C; (2e) trissin; (2f) periviscerokinin (PVK); pyrokinin (PK); and ecdysis-triggering hormone (ETH) (Figure 1).

Cluster 2 contained seven monophyletic clades of GPCRs that included receptors for proctolin, FMRFamide, myoinhibitory peptide (MIP)/sex peptide, and myosuppressin plus three orphan clades (Figure 2). Several of the Cluster 2 clades also contained orphan receptors. Some but not all of the clades generated by our analysis supported previously recognized lineages (16, 18). Key details for the two clusters and sub-assemblages are summarized below.

#### Cluster 1, assemblage 1

##### Sub-assemblage 1a: bursicon, GPA2/GPB5, and ecdysteroid receptors

Our analysis suggests the peptide hormone receptors of sub-assemblage 1b arose from a more recent ancestor of the biogenic amine/steroid hormone GPCRs than other peptide binding GPCRs. We thus refer to these as derived peptide GPCRs. Although we used only the transmembrane region in generating our GPCR phylogenies, all of the derived peptide GPCRs have an extended extracellular N-terminus in common with the leucine-rich repeat-containing G-protein-coupled receptors (LGRs) of which three types are conserved across vertebrates and invertebrates (48). With small exception, the vertebrate LGRs bind the cysteine-rich heterodimer glycoprotein hormones (i.e., GPA/GPB), which regulate gonadal and thyroid activity. This binding affinity is conserved in the dipteran receptors for GPA2/GPB5 (49). Another distinguishing feature of the derived peptide GPCRs is that their ligands are much larger than those of other GPCRs: the largest known ligand for other Class A GPCRs is only 36 amino acids (NPF), whereas the bursicon and GPA/GPB receptors bind ligands that exceed 100 amino acids and form 30 kDa dimers.

Bursicon activates cuticle tanning and hardening shortly after molting by insects (50, 51). Single orthologs of the bursicon receptor (LGR type B) are present in each mosquito and *Drosophila* species (Figure 1). Identification of a GPB ortholog in *D. melanogaster* led to its partner, GPA, and the deorphanization of the GPA2/GPB5 receptor [LGR type A, (49)]. Single orthologs of this receptor are also present in *D. mojavensis* and each mosquito species (Figure 1).

Sub-assemblage 1b also contains a GPCR that binds ecdysteroid hormones, which are major regulators of molting, egg development, and other processes. Most steroid hormones bind a nuclear receptor and potentiate changes in transcription, but some also bind to extracellular receptors including GPCRs (52). Srivastava and colleagues showed that CG18314 from *D. melanogaster* binds dopamine and ecdysteroids, including ecdysone and 20-hydroxyecdysone (53). Binding of dopamine, but not ecdysone, to the receptor leads to the phosphorylation of Akt, a kinase associated with insulin signaling. Ecdysteroid binding activates an extracellular signal-related kinase but no physiological function is currently known for this activity.

We identified five orphan clades (OA1–5) in sub-assemblage 1a (Figure 1). The OA1 and 2 receptors are sister to the bursicon and GPA2/GPB5 receptors and are possible orthologs of the vertebrate relaxin/insulin-like peptide LGRs (OA2). The OA2 orthologs are present in both *Drosophila* species, *Ae. aegypti* and *An. gambiae*, but the OA1 clade appears to have been lost in the Culicidae based on its presence in other insects (data not shown). OA3 is present in all five dipterans, as is OA4. OA5 encodes the *D. melanogaster* gene *moody*, which is implicated in the integrity of the blood-brain barrier (54), although its native ligand is unknown. Our results provide no insights regarding what kind of ligand(s) the OA4–5 orphans bind. However, a previous study that examined small clusters of GPCRs between humans and *D. melanogaster* suggested these orphan clades grouped most closely with peptide receptors (25).

##### Sub-assemblage 1b: CCAP, AKH, corazonin, ACP, and allatotropin receptors

Adipokinetic hormones, corazonin, and CCAP are structurally related but functionally distinct peptides (18). Corazonin and CCAP are cardioactive in *An. gambiae* (55, 56). In *D. melanogaster*, corazonin affects stress resistance and metabolism (57) and is involved in ecdysis, as is CCAP, which is also cardioactive (58). ACP and its receptor were recently identified in insects, including mosquitoes but not in *D. melanogaster*, and its function is unknown (59). Prior studies have also recognized a common evolutionary origin for this receptor group and coevolution of their associated ligands in mosquitoes (18, 59, 60).

Mosquitoes encode two different AKH genes (61) with AKH 1 mobilizing stored carbohydrate but not lipids, and AKH 2 having no effect on these processes or an identified receptor (62). *Drosophila* spp. in contrast encode only AKH 1, which plays a role in starvation-mediated behaviors and mobilization of lipid and carbohydrate stores (58). Recent studies confirm binding of AKH 1 to its predicted receptor in *D. melanogaster* and *An. gambiae*, while also identifying a splice variant receptor in *Ae. aegypti* (61, 63, 64). No AKH receptor was found in *C. quinquefasciatus*.

Allatotropin is not found in *Drosophila*, but in *Ae. aegypti* and other insects it stimulates juvenile hormone (JH) biosynthesis (65). Binding studies identify a single allatotropin receptor in *Ae. aegypti* (AAEL011680) for which a closely related paralog exists (AAEL005310) (65). We also identified one predicted allatotropin receptor in *C. quinquefasciatus* and *An. gambiae*.

#### Cluster 1, assemblage 2

##### Sub-assemblage 2a: sulfakinin related receptors

This sub-assemblage represents the earliest diverging GPCRs in assemblage 2. Sulfakinin regulates feeding behavior in *D. melanogaster* (66) and binds to two GPCR paralogs, CG32540 and CG42301 (67). Both *Ae. aegypti* and *An. gambiae* encode a single sulfakinin ortholog, while *C. quinquefasciatus* encodes two predicted sulfakinin receptor homologs: one that groups with the *Drosophila* sulfakinin receptors and a second that is more closely related to the mosquito sulfakinin orthologs.

##### Sub-assemblage 2b: RYamide, tachykinin, natalisin, NPF, short NPF, SIFamide, and related receptors

The RYamide gene was recently recognized in Diptera and other insects. It encodes two paracopies with no known function that were used to deorphanize a GPCR related to the tachykinin receptor (68, 69). Two copies of this gene are present in mosquitoes. A single gene encodes up to six tachykinin paracopies across the Diptera (13, 18, 70), which are multifunctional (71). *D. melanogaster* was initially thought to encode two tachykinin receptors (CG7887, CG6515) which exhibited preferential binding to different ligand paracopies (72–76). More recent studies show that CG6515 from *D. melanogaster* is not a tachykinin receptor but rather binds natalisin, a newly identified peptide hormone involved in mating (77). *D. mojavensis, An. gambiae*, and *Ae. aegypti* encode a single closely related gene to CG7887, whereas no ortholog is found in *C. quinquefasciatus*. We also detect orthologs of CG6515 in the genome of each mosquito, but the two partial sequences encoded by *Ae. aegypti* (AAEL017414 and AAEL017341) and one paralog in *C. quinquefasciatus* (CPIJ014103) were not long enough to be included in our phylogeny.

Kinins, which were originally identified as myotropic neuropeptides, are now considered key regulators of diuresis in mosquitoes and *Drosophila* (78–81). *D. melanogaster* encodes a single kinin receptor that has been functionally characterized (82). *D. mojavensis, An. gambiae*, and *C. quinquefasciatus* also encode a single predicted kinin receptor, whereas two copies are detected in *Ae. aegypti*. Notably, another type of diuretic peptide, inotocin, the homolog of vertebrate oxytocin/vasopressin, and its receptor are present in locusts and red flour beetles (*T. castaneum*), but both are absent from *Drosophila* spp. and mosquitoes (83).

Our phylogeny indicates the RYamide and tachykinin receptors are related to GPCRs that bind NPF, sNPF, and kinins. The relatedness of the NPF and sNPF receptors is surprising, given differences in ligand structure (36 vs. <11 AA respectively). A prior study also noted the shared homology between insect kinin and vertebrate tachykinin receptors (82). Taken together, we conclude this GPCR receptor cluster is an example of peptide ligand/receptor coevolution in insects.

Neuropeptide F is a member of the NPY/PP/PYY family conserved across vertebrates and higher invertebrates. It was first identified along with its receptor in *D. melanogaster*, where it affects behaviors related to feeding, learning, stress, and alcohol sensitivity (84). The unrelated sNPFs found only in arthropods are encoded by a different gene and processed into four peptides, which affect feeding, metabolism, and growth in *D. melanogaster* (57). The expression of NPF and sNPF is characterized for *Ae. aegypti* and *An. gambiae*, but little is known about their function (85–87). In *Ae. aegypti* but not in other mosquitoes or *Drosophila* spp. there is an apparent duplication of the sNPF gene, which encodes three identical “*Aedes* head peptides” (AHPs) isolated as non- and hydroxylated-proline forms (88, 89). Only the latter inhibits host seeking by females (90) and is also found in the accessory gland of males and transferred to females (91).

The single copy NPF receptor from *D. melanogaster* and one of two NPF copies in *An. gambiae* (AGAP004123) were identified and expressed to demonstrate NPF binding (85, 92). As in *D. melanogaster, D. mojavensis* and *Ae. aegypti* encode a single NPF receptor, whereas *An. gambiae* and *C. quinquefasciatus* encode two. The duplication of the NPF receptors in *An. gambiae* and *C. quinquefasciatus* appears to have occurred independently in each lineage. The two *An. gambiae* NPF receptors, AGAP004123 and AGAP004122, are highly similar at the nucleotide level (99% identical), yet AGAP004122 is truncated relative to AGAP004123 at its 3′ end. The two *C. quinquefasciatus* NPF receptors also exhibit a pattern of high similarity (99%) but truncation of one copy (CPIJ018265) is at the 5′ end. Further study will be needed to determine if these truncated genes are shorter forms of the NPF receptor are incompletely annotated or are pseudogenes.

The first sNPF receptor was identified as a single copy gene in *D. melanogaster* and expressed to confirm binding (93). *An. gambiae* also encodes a single sNPF receptor for which binding was confirmed (85). The structural relatedness of the sNPF and NPF receptors is born out by their shared inhibition of intracellular cAMP signaling (85, 92). Recently, the *Ae. aegypti* sNPF receptor was also demonstrated to bind AHPs, though silencing of the receptor did not impact host-seeking behavior (94).

SIFamide regulates adult courtship behavior in *D. melanogaster* (58), and a SIFamide homolog was previously identified in *Ae. aegypti* (9). A single SIFamide receptor (CG10823) was identified in *D. melanogaster* (95). *An. gambiae* and *D. mojavensis* encode one predicted SIFamide receptor, whereas *Ae. aegypti* and *C. quinquefasciatus* encode two. The SIFamide receptors are most closely related to the four receptors of clade OA8, present only in mosquitoes. Two mosquito receptors from *An. gambiae* and *C. quinquefasciatus* form the orphan clade OA7, which is an exceptionally long branch. Annotation suggests that these may be odorant-binding receptors, although no experimental evidence supports this. OA9 is the outgroup to this large clade of receptors, and is found in all five genomes including duplicate copies in the culicine mosquitoes. Two orphan receptors are found in clade OA6, which forms an outgroup to sub-assemblages 2b–f. These genes reside on a long branch with nothing known about their function.

##### Sub-assemblage 2c: orphan receptors A10 and 11

Two orphan clades, A10 and 11, comprise sub-assemblage 2c. While monophyletic in our analysis, support values for this sub-assemblage are weak. Homologs of A11 are found in all five dipterans, but there is no ortholog of A10 in *C. quinquefasciatus*.

##### Sub-assemblage 2d: CCHamide, allatostatin C and A receptors

All insects appear to encode two CCHamide genes (1 and 2) that arose by duplication after divergence of the hexapods from other arthropods (11, 96). The function of CCHamide 1 and 2 is unknown but binding studies identify two GPCRs from *D. melanogaster* (CG14593 and CG30106) as CCHamide receptors (96). Single copy orthologs of both *D. melanogaster* receptors are found in all species except *C. quinquefasciatus*, which lacks an ortholog of CG14593.

Three structurally distinct types of neuropeptides are called allatostatins in the literature because they inhibit JH synthesis by the corpora allata (CA) in different insects (58, 97). Mosquitoes and *Drosophila* spp. encode: (1) allatostatin A paracopies (FGLamides), (2) allatostatin B paracopies [9–13 AA; herein classified as MIP or Wx(6)Wamides], and (3) a single allatostatin C, also known as PISCF (98–101).

Two allatostatin C receptor paralogs were identified in mosquitoes and *Drosophila* spp. (102, 103) with some studies also suggesting these receptors are related to tachykinin receptors (16, 18) (see below). Our analysis, however, identifies the allatostatin C receptors as a separate clade from the tachykinin receptors, which is consistent with differences in ligand motifs.

Although allatostatin A paracopies inhibit JH biosynthesis in some insects, they have no effect on CA from *D. melanogaster* or female *Ae. aegypti* (104). Recent work suggests a role in foraging behavior of *D. melanogaster* larvae (105). Ast A paracopies bind to two receptor paralogs, CG2872 and CG10001, in *D. melanogaster* (16). We note that *D. mojavensis* and *Ae. aegypti* each encode two receptors for allatostatin A, whereas *An. gambiae* and *C. quinquefasciatus* encode three. The putative allatostatin A receptors from mosquitoes have not been functionally characterized, but their number suggests either all bind one or more allatostatin As or some are orphans.

##### Sub-assemblage 2e: trissin receptor

Trissin is another peptide hormone with no known function that binds a GPCR in *D. melanogaster* (69). We detected single orthologs of the *D. melanogaster* trissin receptor in the other genomes.

##### Sub-assemblage 2f: periviscerokinin, pyrokinin, and ecdysis triggering hormone receptors

Peptide ligands for the receptors in this clade share a similar C-terminal motif (PRL/Vamide) and are processed from precursors encoded by three genes in all dipteran species examined to date. The *capability* gene yields two PVKs ending with PRVamide and one PK ending with WFGPRLamide. The *hugin* gene produces two to four PK paracopies (7, 106) whereas the ETH gene produces two paracopies (9, 18, 58, 60, 107, 108). The receptor for ecdysis-triggering hormone (ETHR) has duplicated in *D. mojavensis*, but exists as a single copy gene in the other genomes.

*Drosophila melanogaster* has three PK GPCRs – CG9918 as the PK1 receptor, and CG8784 and CG8795 as PK2 receptors (109, 110) – and a single PVK receptor (CG14575). Single copy orthologs of the PVK receptor are present in all the species, while variable numbers of PK receptors were found among species. Initially, two were identified in *An. gambiae*, and the expressed CG9918-like receptor (AGAP000658) preferentially binds the PK1 peptides and the other receptor (AGAP003076), the PK2 peptides (108). The same study identified the related receptor in *An. gambiae* as a homolog of the PVK receptor in *D. melanogaster*. Four PK receptors are found in *C. quinquefasciatus*, though only a single PK receptor is found in *Ae. aegypti*.

Both splice variants of ETHR in *D. melanogaster* bind both forms of ETH (18, 60, 100, 107, 108). Both ETH paracopies also regulate ecdysis in *D. melanogaster* and *Ae. aegypti* (60, 107). Two ETHR splice variants were identified in *D. melanogaster* and *Ae. aegypti* that are more selective for ETHs than other related peptides (107, 111–113).

#### Cluster 2

##### Proctolin receptor

Proctolin was the first myostimulatory peptide isolated from an insect, and its receptor was first identified in *D. melanogaster* (58) and an ortholog was identified in *D. mojavensis* (Figure 2). In contrast no gene encoding this peptide or a receptor ortholog is known in mosquitoes, which is consistent with proctolin having no effect on mosquito tissues (114).

##### FMRFamide receptors

The single FMRFamide gene in dipterans encodes multiple paracopies that bind to the expressed *D. melanogaster* GPCR (115, 116) and are myostimulatory in *D. melanogaster* and *An. gambiae* (117, 118). The FMRFamide receptor is represented by a single ortholog in each of the *Drosophila* and mosquito genomes examined (Figure 2).

##### Myoinhibitory/sex peptide receptor

Myoinhibitory peptides were isolated based on their myoinhibitory activity. They were later shown to inhibit JH biosynthesis (so named, allatostatin B) in different insects and to be structurally related to the sex peptides in *Drosophila* (119). Binding studies established that MIPs and sex peptides both bind the *D. melanogaster* MIP receptor (119, 120). A single ortholog of this receptor is also present in *D. mojavensis* and each of the mosquito species (Figure 2). No sex peptide gene is known for mosquitoes, but one study showed that sex peptide from *D. melanogaster* bound to the *Ae. aegypti* MIP receptor (91).

##### Myosuppressin receptors

Myosuppressin inhibits gut and heart contraction in *D. melanogaster* and binds to duplicate GPCRs deorphanized for this species (15, 121). This duplication appears to have occurred prior to the divergence of *D. mojavensis* and *D. melanogaster*. We identified a single ortholog in each mosquito species (Figure 2). Specific binding of myosuppressin was also previously shown for the expressed *An. gambiae* ortholog (23). A related orphan receptor, OA14, is represented as a single ortholog in the *Drosophila, Anopheles*, and *Culex* spp. while two orthologs were identified in *Ae. aegypti* (Figure 2).

##### Other orphan clades

In addition to OA14, Cluster 2 contains two other orphan clades designated as OA12 and 13. OA12 is most closely related to the proctolin receptor but has expanded in the culicine mosquitoes, with *Ae. aegypti* encoding three copies and *C. quinquefasciatus* encoding four. OA13 has an uncertain position in Cluster 2 but at least one copy of this gene is found in each of the genomes with the exception of *An. gambiae*.

### Class B secretin GPCRs

The balance of dipteran GPCRs that bind peptide hormones belong to the Class B secretin family (7). Our analysis distinguished nine clades within this class (Table 1; Figure 3), of which three have identified ligands: (1) calcitonin-like diuretic hormone (CT-DH), (2) corticotropin-releasing factor-like diuretic hormone (CRF-DH), and (3) pigment dispersing factor (PDF) (Figure 3). We included the latrophilin GPCRs that are conserved across invertebrates and vertebrates (122, 123) and bind latrotoxins from spiders in the genus *Latrodectus* (124). The endogenous ligand for the vertebrate receptors is teneurin-2, a glycoprotein displayed on the surface of cells (125), however the endogenous ligand in insects in unknown. Class B secretin GPCRs also include the *Methuselah* and *Methuselah-like* genes, which are grouped into four classes, A–D, based on the disulfide bridges present in their extracellular domain (126). Other phylogenetic data for Class B GPCRs have been produced for vertebrates and invertebrates (127) and insects alone (126).

#### Pigment dispersing factor receptor

This peptide was first identified by its stimulation of pigment dispersal in some arthropods, but in insects PDF was the first neuropeptide shown to regulate circadian activity in *D. melanogaster* (58). Its receptor was subsequently identified, and we found single orthologs of this receptor in *D. mojavensis* and each of the mosquito species. It is not known whether PDF has a role in circadian activity in insects generally, but host-seeking behavior is one circadian behavior that may be regulated by PDF in mosquitoes (128).

#### Corticotropin-releasing factor-like diuretic hormone receptor

Our phylogeny suggests the ancestor of this receptor underwent a duplication event prior to the divergence of the Culicidae and Drosophilidae. Both orthologs from *D. melanogaster* bind CRF-DH (129). The diuretic activity of CRF-DH is also well characterized in mosquitoes and *D. melanogaster* (130, 131).

#### Calcitonin-like diuretic hormone receptor

These ligands and their associated receptors mediate diuresis by Malpighian tubules (132). The activity and expression of CT-DH and its receptor are well characterized in mosquitoes and *D. melanogaster* (132–134). The CT-DH receptors are single copy in each genome and are sister to the orphan Hector-like GPCRs (Figure 3).

#### Methuselah-like receptors

Studies in *D. melanogaster* described mutations in a GPCR named Methuselah (*mth*), which conferred increased longevity and stress resistance (135). Additional *mth-*like genes were thereafter identified (34), while genome studies indicate that *mth* genes are likely present in all insects. Four major groups (A–D) of Mth receptors have been proposed on the basis of the disulfide bridges present in their extracellular domains (126, 136). While present in all insects examined in this study, *D. melanogaster* encodes many more *mth*-like genes (16) than *D. mojavensis* (8), *C. quinquefasciatus* (8), *Ae. aegypti* (6), or *An. gambiae* (4). We further note the *Drosophila* Mth receptors are widely distributed across the tree and are not monophyletic. Only three clades of Mth receptors include members from the Culicidae: the C type Mth, Mth-like 14, and a clade that is sister to the B and D type Mth receptors (Figure 3). Cvejic et al. (137) reported that the endogenous ligand for the Mth receptor in *D. melanogaster* was a peptide encoded by the gene *stunted*, and that disruption of its expression has a similar phenotypic effect as reduced *mth* expression. Others, however, report that Mth binds *Drosophila* sex peptide and other synthetic peptides and suggest this receptor may be a promiscuous GPCR with diverse ligands (138).

#### Latrophilin receptor

Each genome contains a single copy of the latrophilin GPCR. Our analysis suggests these receptors do not form a monophyletic group in the Diptera, but this conclusion is based on poor alignment due to the loss of six of the seven transmembrane domains in the Culicidae. The mosquito receptors, however, retain N-terminal domains that are similar to those of *Drosophila* spp. (data not shown). Truncation of the C-terminal region of latrotoxin-binding GPCRs in mammals does not impede responses to latrotoxin in cell culture (139), which suggests the culicid orthologs may be functional.

#### Orphan Class B/secretin receptors

In addition to the Mth-like and latrophilin GPCRs, our results identify two clades of orphan Class B secretin GPCRs. The Hector group (OB1) is sister to the CT-DH receptor and appears to have arisen by a duplication event in Diptera that predates the divergence of the Drosophilidae from the Culicidae. Although orphans, mutations in the Hector GPCRs have been shown affect mating behavior in *D. melanogaster* (140). OB2 was identified as a distant ortholog of the human epididymis six GPCR, itself an orphan, shortly after the publication of the *D. melanogaster* genome (34).

### Receptor guanylyl cyclases

**Figure 4:**
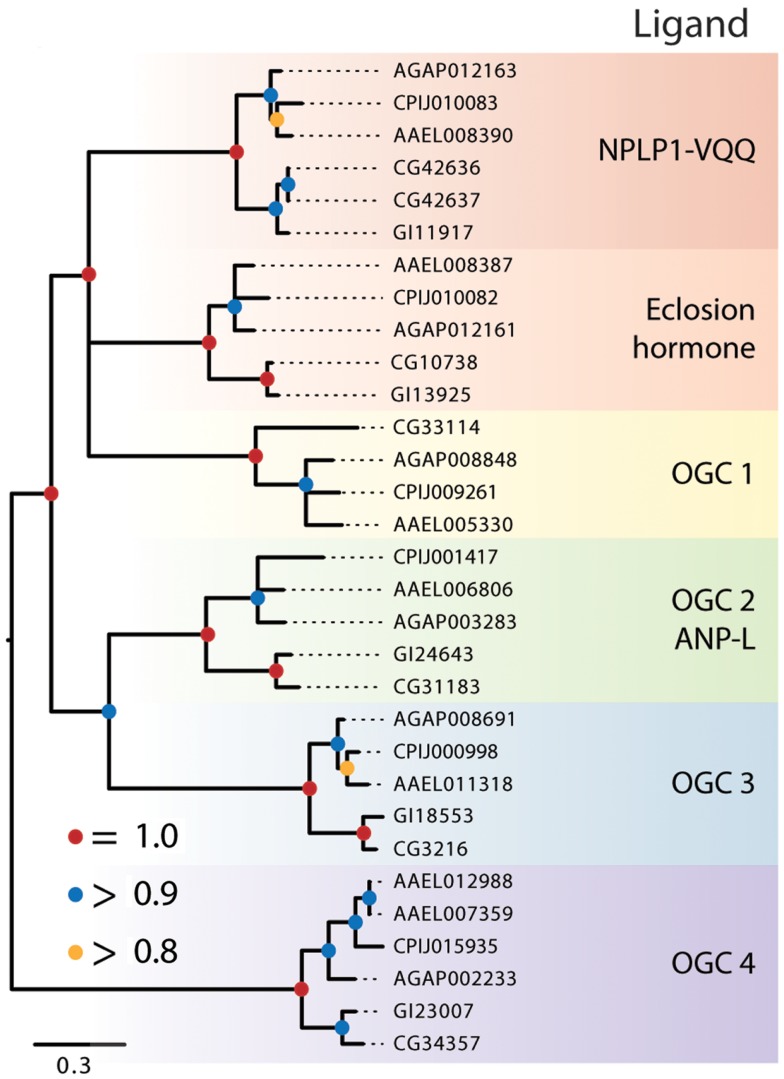
**Maximum-likelihood tree of membrane-bound RGCs using the five dipteran species described in Figure 2 with alignments made using the protein kinase domain of each receptor**. Branches with maximum-likelihood support values <0.8 have been collapsed to polytomies. The tree was rooted at the midpoint.

Receptor guanylyl cyclases are conserved homodimeric membrane proteins (~200 kDa) with intracellular protein kinase and guanylyl cyclase domains that catalyze cGMP formation (2). We identified six RGC clades in Diptera (Figure 4), two of which have characterized ligands: eclosion hormone (EH) and neuropeptide-like peptide 1-VQQ (NPLP1). Eleven forms of NPLP1 are encoded by the genes identified for *D. melanogaster* and other insects (141). The NPLP1 receptor was also shown to be an RGC in *D. melanogaster*, while functional studies showed that NPLP1 stimulates fluid transport in the midgut and Malpighian tubules, modulates stress, and affects immune responses (142). EH is a co-activator with ETH of ecdysis behavior in insects. Expression of the *Drosophila* EH receptor in a mammalian cell line followed by incubation with EH from *D. melanogaster* or another EH paracopy from the oriental fruit fly (*Bactrocera dorsalis*) elicited a strong cGMP response, which provided evidence this RGC functions as an EH receptor (143).

The remaining dipteran RGCs resided in orphan clades, and to further characterize their relationships to other RGCs, we constructed a phylogenetic tree that included these RGCs and the five RGCs from *H. sapiens*, A–E (data not shown). This analysis indicated that two of the dipteran orphan clades (OGC1 and 3) have no vertebrate ortholog. In contrast, OGC2 appears orthologous to vertebrate RGC-A and -B that bind natriuretic peptides, while OGC3 is homologous to the vertebrate retinal RGC. Overall, our analysis indicates that dipteran RGCs have undergone few changes, with the possible exceptions of an apparent loss of OGC1 from *D. mojavensis*, the duplication of the NPLP receptor in *D. melanogaster*, and the duplication of the OGC4 homolog in *Ae. aegypti*.

### Protein kinase receptors

**Figure 5:**
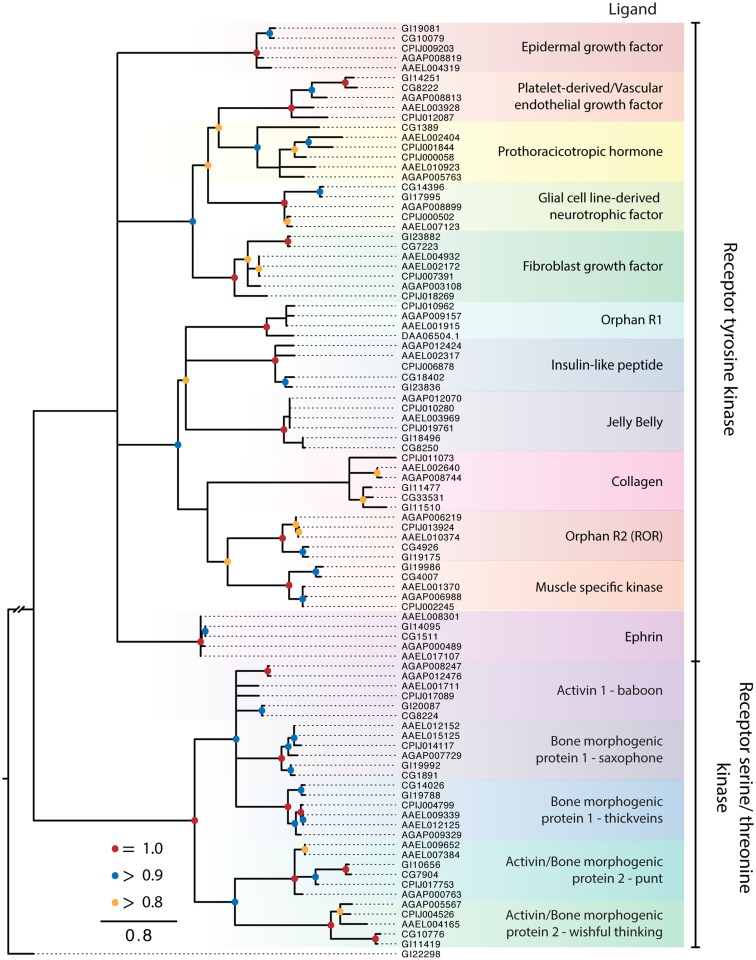
**Maximum-likelihood tree of PKRs using the protein kinase domains of PKRs from the five dipteran species described in Figure 2**. The tree was rooted using a guanylyl cyclase receptor GI22298 from *D. mojavensis*. Branches with maximum-likelihood support values <0.8 have been collapsed to polytomies. The *C. quinquefasciatus* gene CPIJ006878 has been placed within the clade it presumably belongs to, though it lacks the protein kinase region used for alignment. The *D. mojavensis* OR1 ortholog is not annotated in FlyBase, but EST evidence supports its presence (160). Clades are named after their characterized ligands, if known.

Protein kinase receptors form non-covalently bound dimers upon reaching the cell surface or in response to ligand binding (2). The insulin receptor is an exception in that its monomers undergo proteolysis into α and β subunits that are linked by intra- and inter-subunit disulfide bonds to form a covalent stabilized heterotetramer (144). Peptide hormones, growth factors, and membrane proteins are ligands for these receptors, and their interactions, signaling, and function are well characterized for mammalian systems, intermediately characterized for *D. melanogaster*, and poorly characterized in other insects. Ligand binding to specific extracellular domains activates intracellular kinase domains that autophosphorylate either tyrosine or serine and threonine residues, presenting docking sites for cytoplasmic effectors, adaptors, and scaffold proteins that in turn activate one or more signal pathways (3). Activated PKRs are also targets of protein phosphatases that modulate and even block signaling in mammals. Our analysis of dipteran PKRs produced a phylogeny with two major branches: (1) an assemblage of 12 clades consisting of primarily characterized receptor tyrosine kinases (RTKs) and (2) five clades related to mammalian transforming growth factor beta (TGF-β) receptors, which contain a distinctive serine-threonine kinase domain (Figure 5). Similar to the RGCs but unlike the GPCRs, dipteran PKRs have undergone relatively few lineage-specific gains or losses, suggesting that most members were likely present in the most recent common ancestor of culicids and drosophilids.

#### Receptor tyrosine kinases

The RTK clades we identified were conserved across the five dipteran species (Figure 5). Ten of these clades are also represented among the 20 recognized subfamilies of human RTKs, which have been classified on the basis of ligand interactions and other features (3). The remaining two RTK clades are known only from insects and other arthropods. One of these is an orphan receptor (OR1), which contains a venus flytrap domain, while the other is the prothoracicotropic hormone (PTTH) receptor. Most of the dipteran RTK clades are encoded by a single ortholog in each species. For the 10 clades with apparent human homologs, only OR2 (ROR) is an orphan. Five others interact with secreted ligands [epidermal growth factor (EGF), platelet-derived/vascular endothelial growth factor, fibroblast growth factor (FGF), insulin-like peptides (ILPs), and low-density lipoprotein repeat-containing factor], whereas the remaining four form activating complexes with unrelated membrane proteins (glial cell line-derived neurotrophic factors, collagen, muscle specific kinase, and ephrin).

##### ILP and related receptors

The ILP receptor (IR) was the first RTK identified in insects when cloned from *D. melanogaster* (145, 146). These studies showed this RTK bound mammalian insulin, and much later, the discovery of multiple ILP genes in *D. melanogaster* was taken as evidence that the encoded peptides were the endogenous ligands of the IR (147). Up to eight ILPs encoded by different genes are known for the dipteran species in our study (148), and all but the ILP6 subfamily are likely processed into disulfide-linked dimers (6–8 kDa). Only one study from *Ae. aegypti* confirms the high affinity binding of an endogenous insect ILP to its cognate IR (149), while another shows ILP displacement of human insulin bound to the *D. melanogaster* IR (150). Several studies also show the IR activates the canonical insulin signaling pathway in *Ae. aegypti* (151–153) and that ILPs have a diversity of functions in mosquitoes (148, 154).

The two RTKs that group with the IR include a homolog of human anaplastic lymphoma RTK (ALK) and leukocyte tyrosine kinase (LTK), and the orphan OR1. Both ALK and LTK play key roles in human cancers, whereas the ALK homolog in *D. melanogaster* directs gut and nervous system development and is associated with ethanol sensitivity and learning (155–158). No specific ligand has been identified for mammalian ALK or LTK, although a 61 kDa secreted protein ligand named Jelly Belly has been identified as an ALK ligand in *D. melanogaster* (159). However, Jelly Belly does not interact with mammalian ALK (159). Our analysis supports the presence of OR1 in mosquitoes, other insects, and other invertebrates as well as its loss from the *D. melanogaster* subgroup as first reported by Ahier et al. (160). Curated, partial mRNAs indicate this receptor is present in *D. mojavensis*, which was included in the phylogenetic tree, but the genome lacks an annotated receptor. The extracellular domain of OR1 contains a venus flytrap domain, which is also found in some GPCRs and RGCs, and has been implicated in binding amino acids and other small molecules (160, 161).

##### PTTH receptor

Prothoracicotropic hormone regulates molting and development in insects by stimulating the prothoracic glands (PGs) to produce ecdysteroid hormones (162–164). Expression of the RTK Torso is required for PTTH action in *D. melanogaster*. Torso was also shown to interact with Trunk, which is a protein growth factor that regulates early embryonic development. PTTH and Trunk are structurally related to other protein and growth factors that contain a cysteine knot motif and bind RTKs and TGF-β receptors. Although Trunk and PTTH differ in tissue and temporal expression, their interaction with Torso activates the Ras/mitogen-activated protein kinase pathway and calcium signaling. These shared features and actions support the designation of Torso as the PTTH receptor. However, direct binding of PTTH to Torso has not been demonstrated.

We identified duplicate PTTH RTK genes in *C. quinquefasciatus* and *Ae. aegypti*, but not in *An. gambiae* or *D. melanogaster*. The absence of a PTTH RTK ortholog in *D. mojavensis* is likely due to our inability to identify it rather than its loss given that orthologs of the *trunk* and *PTTH* genes are present (163). PTTH expression has been profiled in mosquito larvae (165–167), but it is not known to regulate ecdysteroid production, which occurs in unidentified cells in the abdominal or thoracic wall, not the PGs (168).

##### Other RTKs

The functional significance and signaling of the other RTK clades has been examined in *D. melanogaster*. The best characterized of them is the EGF receptor, which interacts with one secreted ligand (Vein) plus three other ligands (Spitz, Keren, and Gurken) derived from enzymatic cleavage of inactive membrane-bound precursors. Typically, these ligands regulate the trajectory of specific embryonic and tissue stem cell types (169, 170). An EGF receptor exists in each mosquito species, and its expression has been characterized in *An. gambiae* (171). However, the putative ortholog in *C. quinquefasciatus* was a partial annotation that lacked the protein kinase domain and was therefore not included in the phylogeny. The platelet-derived/vascular endothelial growth factor receptor and its three secreted ligands are involved in the regulation of hemocyte and midgut stem cell fate in *D. melanogaster* (172, 173). The FGF receptor is duplicated in each dipteran species. Functional studies in *D. melanogaster* show the two receptors differ in their structure and ligand interactions (174). Homolog 1 (Heartless, CG7223) interacts only with two of the three related secreted FGFs (Pyramus and Thisbe) to direct mesoderm migration and heart muscle differentiation of the dorsal vessel, whereas homolog 2 (Breathless, CG32134) is activated by its ligand (Branchless) to define branching of the tracheal system. Breathless appears to be incompletely annotated, and was removed from our alignments. Less is known about the RET RTK (175, 176), ROR RTK (177), and ephrin RTK (178) in the development of *D. melanogaster*, while the collagen and muscle specific kinase RTKs remain unstudied. We note that *D. mojavensis* encodes duplicate collagen receptors, while the *D. melanogaster* paralog CG34380 lacks the protein kinase domain used for alignment.

#### TGF-β-like receptors

The different elements of the TGF-β signaling pathway are well characterized in mammals and *D. melanogaster*, because they regulate many of the same developmental and cellular processes as RTKs (179–181). In common with secreted RTK ligands, TGF-β ligands are disulfide-linked homodimers (~30 kDa) of N-terminal regions cleaved from precursors. They are subdivided into the TGF-β/activin orthologs, Activin, Dawdle, Myoglianin, and Maverick, and bone morphogenic protein (BMP) orthologs, Decapentaplegic, Glass Bottom Boat/60A, and Screw, in *Drosophila* (182) and *Anopheles* spp. (183). In general, these ligands act as growth factors and cytokines in both mammals and insects (182).

Types I and II TGF-β receptors are single pass-transmembrane serine/threonine kinases, and ligand binding results in the formation of a heterotetramer of types I and II receptor dimers with the type II dimer phosphorylating the type I dimer, which in turn binds and phosphorylates SMAD or MAD proteins that activate other elements in a signaling pathway. In agreement with these studies, we identified three type I receptor clades (Baboon, Saxophone, Thickveins) and two type II receptor clades (Punt and Wishful Thinking). Each clade is represented as a single ortholog in the two *Drosophila* spp. and *C. quinquefasciatus*. Duplications have arisen in *Ae. aegypti* in the *saxophone, thickveins*, and *punt* genes. In contrast, *An. gambiae* has paralog copies of the *baboon* receptor.

The TGF-β ligands are not considered peptide hormones, *per se*, but activin may be an exception in that it is widely expressed in the nervous system and in neurosecretory cells associated with endocrine glands in *D. melanogaster* (182, 184). Mammalian activin homologs are also expressed in neuroendocrine cells in the pituitary gland, where they regulate the expression and release of gonadotropic hormones (185), and in endocrine cells in the pancreas islets, where they modulate insulin secretion (186). Other studies show that TGF-β signaling plays an important role in mosquito immunity and is responsive to mammalian TGF-β proteins ingested in blood meals (187, 188).

### Conservation of active sites in receptors and hormones

**Figure 6:**
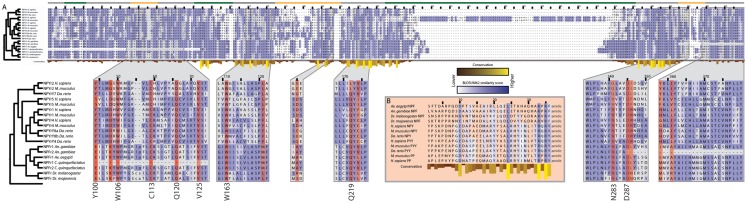
**Conservation of peptide active sites and receptor binding regions across the Bilateria**. **(A)** Alignment of neuropeptide Y GPCRs subtypes (NPYr) that also bind pancreatic peptide (PP) and peptide YY (PYY) from *M. musculus, Da. rerio*, and *H. sapiens* with neuropeptide F GPCRs (NPFr) from the five dipteran genomes. Blue shading indicates amino acid similarity between residues according to BLOSUM62 scores; darker shades indicate increased similarity. Yellow bars below residues indicate conservation across the alignment. Position relative to the cell membrane is indicated by the colored line above the alignment: intracellular: green, extracellular: yellow, transmembrane: gray. Zoomed regions show domains that have been confirmed experimentally to be important for ligand binding by NPYrs. Residues highlighted in red indicate those determined to be involved in ligand binding in *H. sapiens* NPYr1 and 2. Binding residues that are highly conserved are listed next to the alignment with their amino acid in human NPYr1 or 2. **(B)** Alignment of vertebrate NPY, PP, and PYY with dipteran NPF sequences. Only the mature peptide is shown. Coloring is the same as in **(A)**.

An underlying assumption in the study of peptide hormone receptor evolution is that related receptors exhibit a degree of conservation in their active site, as do their peptide ligands. To test this, we compared the sequences of related NPFs and their GPCRs for the five dipterans to the NPY/PP/PYYs and their receptors in three vertebrates: zebrafish (*D. rerio*), mouse (*M. musculus*), and human (*H. sapiens*) (Figure 6). Insect NPF is a member of the NPY family, and evidence suggests that the neuropeptides regulate related behaviors across these groups (58).

Mutagenesis studies have revealed a number of receptor residues that are involved in NPY binding (189, 190). We examined the corresponding locations in the dipteran receptors and found that many key binding residues are conserved, including several that are perfectly conserved, since the divergence of protostomes and deuterostomes 650 Mya. Loops 2–4 comprise a hydrophobic ligand binding pocket in NPY receptors (191), and within this pocket, multiple residues have been implicated in ligand-receptor interactions (41–43, 192, 193) (Figure 6). Six of the residues identified by mutagenesis of human NPY receptors are perfectly conserved in the full-length dipteran NPF receptors: W106, C113, Q120, V125, W163 and D287 (numbering refers to human NPYr1/2 positions). The first four residues are absent in the N-terminal truncated *C. quinquefasciatus* copy of the NPF receptor, while D287 is absent in the C-terminal truncated *An. gambiae* NPF receptor. Q219 is conserved in all receptors examined except *H. sapiens* and *M. musculus* NPYr1. Similarly, N283 is invariant in the dipteran NPF receptors, but differs between vertebrate forms of the receptor. The hydroxyl group of Y100 is implicated in NPY binding by the NPYr1 from *H. sapiens* (192). In the dipteran receptors, the homologous position is invariantly a glutamic acid with the exception of the truncated *C. quinquefasciatus* receptor, where it is absent. Although the amino acid at this position is different between the vertebrate and dipteran lineages, the glutamic acid residue still contains a free hydroxyl group, suggesting that the functionality of this position is conserved.

Among the NPF/NPY family, 3 of 36 residues in the mature peptide are perfectly conserved in the species examined: L24, R33, and R35 (Figure 6). The C-terminal A/TRXRY/Famide motif along with a D/E at position 10, and a leucine residue at position 24 of the mature peptide were also conserved. These data overall strongly suggested functional sites are conserved between related but distinct peptides and their associated receptors across hundreds of millions of years of evolution.

## Conclusion

This study represents the first comprehensive analysis of the three receptor types that bind peptide hormones and growth factors in insects. Our primary motivation for undertaking this study was to develop a robust phylogenetic framework to study the function of particular orphan receptors in mosquitoes. However, by taking advantage of the comparative genomic data available for the Culicidae and Drosophilidae, our results also provide comprehensive phylogenetic information for the GPCRs, RGCs, and PKRs across the breadth of the Diptera (Nematocera and Brachycera).

The absence of any individual orphans within the clades of RGCs with characterized ligands suggests these receptors are stable within the Diptera and that diversification of RGCs occurred prior to the evolution of the order several hundred million years ago (44). Dipteran Class A and B GPCRs are more evolutionarily labile with each experiencing several instances of duplication and loss. For Class A GPCRs, most of the orphans in characterized clades are single duplication events that have occurred in a particular mosquito species. The functional significance of these duplications is in most cases unclear. Since many peptide hormones exist in multiple forms derived from a single propeptide, we speculate that different forms of a given receptor may preferentially interact with different forms of their cognate peptide hormone. In some cases, paralog receptors both bind a single hormone but vary in their binding affinity and phenotypic effect, as is seen in the two PK2 GPCRs of *D. melanogaster* (194). On the other hand, most of the GPCR orphan clades are Methuselah-like Class B secretin GPCRs, which are overrepresented in drosophilids generally and *D. melanogaster* in particular. Currently, the literature offers no insights into why this bias exists or what the functional significance of so many Methuselah-like GPCRs might be.

We tried to characterize the evolution of gains and losses of peptide hormone receptors in the Diptera but in many cases it was not possible to discern such events with certainty due to issues with annotation. Often what initially appeared as a gene duplication event was in fact two separate gene annotations for a single gene. This was verified in some cases by available RNAseq data covering the genes of interest, whereas in others, alignment to a single ortholog demonstrated that the actual gene had been divided during gene prediction. This was particularly true in the Class A rhodopsin-like GPCRs of Cluster 1. We had to omit some GPCRs from the analyses due to absence of the domain needed for identification and alignment. Several potential PKRs also lacked a complete kinase domain and were therefore removed from our analysis. Though care was taken to correct these annotation errors in our analysis, it is possible that some duplication events we report are artifacts of gene prediction algorithms.

Studies across a range of organisms have shown the utility of comparing orphan to characterized receptors. Hansen and colleagues (59) demonstrated that an orphan GPCR was sister to the AKH receptor, and these two receptors were sister to the corazonin receptor. Subsequent efforts to deorphanize the receptor identified a structurally intermediate peptide encoded in the genome of insects. Our results illuminate several orphan clades and individual receptors that are sister to characterized receptors, suggesting that these orphans may bind similar ligands. Additionally, the species distribution of orphan receptors and patterns of tissue and temporal expression may also reduce the target ligand pool, and hopefully aid in deorphanization. While GPCRs evolve at a fast rate (195), binding sites in both the receptor and peptide ligand can be conserved across hundreds of millions of years as demonstrated here for the vertebrate NPY/PP/PYY and dipteran NPFs and their GPCRs. Conservation of functional residues between receptors that bind related peptides suggest that phylogenetic position of related orphan receptors can aid the identification of ligands and provide insights into their function, because in many instances it is also conserved to a high degree, as demonstrated for the NPF/NPY superfamily (84). Thus, our results could assist in deorphanizing receptors in newly sequenced mosquito genomes by identifying their ligands. On the other hand, the literature is more equivocal in regard to sequence similarity between species also resulting in functional similarity. Thus, in addition to deorphanization, considerable work remains in understanding the physiological function of many peptide hormones and growth factors among different species of mosquitoes and other insects.

## Author Contributions

Kevin J. Vogel, Mark R. Brown, and Michael R. Strand designed the study, Kevin J. Vogel performed the analyses, and Kevin J. Vogel, Mark R. Brown, and Michael R. Strand wrote the manuscript.

## Conflict of Interest Statement

The authors declare that the research was conducted in the absence of any commercial or financial relationships that could be construed as a potential conflict of interest.

## Supplementary Material

The Supplementary Material for this article can be found online at http://www.frontiersin.org/Journal/10.3389/fendo.2013.00193/abstract
